# Clinical magnetic resonance-enabled characterization of mono-iodoacetate-induced osteoarthritis in a large animal species

**DOI:** 10.1371/journal.pone.0201673

**Published:** 2018-08-03

**Authors:** Mark D. Unger, Naveen S. Murthy, Rahul Kanwar, Kasey A. Strand, Timothy P. Maus, Andreas S. Beutler

**Affiliations:** 1 Departments of Anesthesiology and Oncology, Mayo Clinic, Translational Science Track, Mayo Graduate School, Rochester, MN, United States of America; 2 Department of Radiology (Section of Interventional Pain Management), Mayo Clinic, Rochester, MN, United States of America; Drexel University, UNITED STATES

## Abstract

**Introduction:**

Osteoarthritis (OA) is the most common form of arthritis. Medical and surgical treatments have yet to substantially diminish the global health and economic burden of OA. Due to recent advances in clinical imaging, including magnetic resonance imaging (MRI), a correlation has been established between structural joint damage and OA-related pain and disability. Existing preclinical animal models of OA are useful tools but each suffers specific roadblocks when translating structural MRI data to humans. Intraarticular injection of mono-iodoacetate (MIA) is a reliable, well-studied method to induce OA in small animals but joint size discrepancy precludes the use of clinical grade MRI to study structural disease. The porcine knee is suited for clinical MRI and demonstrates homology with humans. We set out to establish the first large animal model of MIA-induced knee OA in swine characterized by structural MRI.

**Materials and methods:**

Yucatan swine (n = 27) underwent ultrasound-guided injection of knees with 1.2, 4, 12, or 40 mg MIA. MRI was performed at several time points over 12 weeks (n = 54 knees) and images were assessed according to a modified clinical grading scheme. Knees were harvested and graded up to 35 weeks after injection.

**Results:**

MIA-injected knees (n = 25) but not control knees (n = 29) developed gross degeneration. A total of n = 6,000 MRI measurements were recorded by two radiologists. MRI revealed progressive cartilage damage, bone marrow edema, erosions, and effusions in MIA-injected knees. Lesion severity and progression was influenced by time, dose, and inter-individual variability.

**Conclusions:**

Intraarticular injection of MIA produced structural knee degradation that was reliably characterized using clinical MRI in swine. Destruction was progressive and, similar to human OA, lesion severity was heterogeneous between and within treatment groups.

## Introduction

The most common form of arthritis is osteoarthritis (OA), a chronic disease distinguished by progressive, structural joint damage[1–3]. OA is the leading cause of disability due to joint compromise[4,5] affecting over 240 million people around the world annually[6,7]. In patients over 50, knee OA is the most common cause of lower limb disability[8]. Available treatment options are largely inadequate, as evidenced by the rise in total knee replacements performed each year[2,6]. At least $28 billion in hospital costs related to knee replacement were accrued by patients in the United States during 2009[7,9–11]. A net reduction in OA-related disability has not been realized[1–8,12].

Animal models are critical to the future development of new treatment options for knee OA. Most animal testing of OA has been done in rodents[5,13–17]. More advanced models that are good approximations of humans in terms of knee anatomy, cartilage structure, and body weight have also been reported using large animals such as dogs[18–21]. While dogs have many properties favorable for use in OA models, interest in replacing companion animals with use species (in the research setting) has led to the development of knee OA models using swine, which offer a similarly faithful anatomical representation of the human knee[22–28]. However, existing swine models suffer from some critical shortcomings that limit their utility especially when quantitative results are desired as outcome parameters such as grading the benefit of a (hypothetical) new OA treatment[23,27]. An ideal large animal knee OA model should i) allow for efficient induction of the pathology such as by a simple intraarticular injection, ii) rely only on a widely available, well-standardized use animal species such as swine, iii) allow the induction of several states of severity (such as “mild,” “moderate,” and “severe”), and, most importantly, iv) provide an *in vivo* method for the quantification of OA knee damage that allows for serial assessment in the course of a study without necessitating the sacrifice of test subjects at (inefficiently) early time points.

Clinical imaging technology has enhanced our understanding of the relationship between structural pathology and OA disease status[29–32]. Magnetic resonance imaging (MRI) was recently identified by the Osteoarthritis Research Society International as the most appropriate method to evaluate joint status in OA research[32]. This recommendation comes after several studies reported a correlation between anatomically specific MRI findings and OA-related pain[33–40]. For instance, bone marrow lesions, synovitis, effusion, cartilage degradation, and bone attrition were associated with clinical findings more frequently than damage to menisci, osteophytes, or periarticular lesions[29–31,41].

Here we report a knee OA model in Yucatan swine induced by intraarticular mono-iodoacetate (MIA). Clinical grade, 3 Tesla (T) MRI was employed for a comprehensive assessment of MIA induced joint damage and for serial quantification of lesions characterizing the evolution of OA over time. Four dose levels of MIA were tested in a total of n = 27 swine, assessing n = 54 knees for up to three times generating n = 6,000 data points acquired by blinded assessment of MRI images by two board certified specialty radiologists. Multivariate analysis of MRI results and correlation with pathologic knee specimens generated a comprehensive quantitative characterization of the capabilities and limitations of this new model with utility for the future design of preclinical knee OA studies in swine.

## Materials and methods

### Animals

This study was carried out in strict accordance with the recommendations in the Guide for the Care and Use of Laboratory Animals of the National Institutes of Health. The protocol was approved by the Mayo Institutional Animal Care and Use Committee (Protocol Numbers: A33715 and A212716). All study personnel involved in animal use were accredited by the Mayo Department of Comparative Medicine and all efforts were made to minimize suffering. Between 01/2016 and 01/2018, a total of n = 27 animals were used. Animals were monitored by veterinary staff on a daily basis and no animal required premature euthanasia to limit suffering. All animals were euthanized according to the planned experimental endpoint which was experiment specific (S1 Table). The MIA dose range tested was determined through pilot testing followed by serial addition of intermediate and low MIA dose cohorts to arrive at a useful pre-clinical model of large animal OA. Starting doses were determined by scaling doses used in other species according to estimated body surface area [5,17]. 1.2, 4, 12, or 40 mg MIA dissolved in 2 or 3 ml phosphate buffered saline (PBS) was injected into one hindlimb knee joint. The contralateral knee received either diluent only (sham injection) or no injection. S1 Table summarizes the characteristics of animals used, knee treatment conditions, and duration of experiments.

### MIA injection

In a laminar flow hood two hours prior to knee injection, MIA (Sigma) was dissolved in sterile, pH 7.4 PBS, vortexed, sterile filtered, and drawn into sterile syringes, which were then labeled with a blinding code by a member of another laboratory. Injections were performed by a musculoskeletal radiologist (NSM) blinded to the injectate and the side of MIA injection was randomized. All procedures were performed under general anesthesia and strict aseptic technique. Anesthesia was induced by intramuscular tiletamine/zolazepam (Telazol), xylazine, and glycopyrrolate. Animals were then intubated and anesthesia was maintained by isoflurane (1.5–2.5%, titrated to effect) in 50% O_2_. Animals were monitored continuously and health status was documented at least every 15 minutes for heart rate, respiration rate, body temperature, ventilation, oxygenation, and depth of anesthesia by eliciting reflex responses. Skin over both knee joints was surgically clipped and scrubbed with 1% chlorhexidine. A 23-gauge needle was inserted from lateral to medial under ultrasound guidance into the intraarticular, synovial space between the patella and trochlea. The patellofemoral articulation was identified by the interface between hyperechoic cortical bone, representing the trochlea of the femoral condyle, and anechoic synovial fluid contained within the intraarticular space. This interface allowed clear delineation of the hyperechoic needle tip in real time. Intraarticular injections were performed at this location during week 0 of the study. A 100 μl PBS test injection confirmed dispersion of injectate in the synovial space. Animals that received 3 ml total volume of injectate were also subjected to passive knee flexion and extension immediately after withdrawing the needle to distribute the injectate.

### MR imaging

Swine were anesthetized, intubated, and monitored as described above for imaging. Animals were scanned in a lateral decubitus position with the rear legs bolstered in an extended position (S1 Fig). A custom 8 channel surface coil was wrapped around both rear legs in a 3T imager (GE Healthcare). Fat-saturated proton density images (TE 18, TR 2000, ETL 4,384 x 256 matrix, 3 mm slice thickness, 0.5 mm spacing, 3 NEX) were performed in sagittal, axial (perpendicular to the patellofemoral joint), and coronal (parallel to the patellofemoral joint) planes. A 3D SPGR acquisition was also obtained (flip angle 10, 320 x 192, 1.5 NEX, slice thickness 1 mm). S2 Fig outlines the MRI time points obtained for each animal.

### Grading of MR images

Swine knee images were graded by a musculoskeletal radiologist (NSM) and neuroradiologist (TPM) (combined experience over 55 years) blinded to knee treatment status using an adaptation of the International Cartilage Repair Society clinical grading system[42]. Each knee joint was partitioned into 6 joint surfaces (medial femur, lateral femur, medial tibia, lateral tibia, trochlea, and patella). Each joint surface was assessed for cartilage integrity (grade 0: normal thickness and appearance, grade 1: nearly normal appearance with superficial indentation, grade 2: cartilage thinning of less than 50%, grade 3: cartilage thinning from 50% up to 100% but not including erosion of underlying subchondral bone, or grade 4: complete loss of cartilage with associated damage to underlying subchondral bone), bone marrow edema (grade 0: normal, grade 1: small hyperintense foci extending from inner margin of cartilage into subjacent subchondral bone, grade 2: moderate extension of hyperintense foci, or grade 3: large extension of hyperintense foci), and erosions (absent: 0 or present: 1). The entire knee was evaluated for severity of joint effusion (grade 0: no effusion, grade 1: small sized effusion, grade 2: moderate sized effusion, or grade 3: large sized effusion). The integrity of the menisci, cruciate ligaments, collateral ligaments, quadriceps, and patellar tendons were also assessed (intact: 0 or damaged: 1). Heat maps were generated to summarize lesion severity from a total of n = 4,560 unique MRI measurements in four categories (cartilage damage, bone marrow edema, subchondral erosion, and joint effusion). Each measurement was divided by the maximum respective grade possible to yield an absolute value. At each MRI time point n = 19 data points per knee were measured, including n = 6 areas for articular cartilage, bone marrow edema, and erosions. The entire knee was evaluated at once for evidence of joint effusion. A total of n = 12 knees were evaluated at n = 3 successive time points and n = 42 knees were evaluated at n = 2 successive time points. Thus, [(12 knees x 3 time points x 19 anatomical locations of measure) + (42 knees x 2 time points x 19 anatomical locations of measure) x 2 observers] = 4,560 unique data points. Paired measurements for each data point from two observers were combined by taking the median. These values were grouped according to their anatomical location, time point, and treatment group and a median was calculated at each of the n = 19 points of measure. This second median was taken as the absolute grade for that respective damage category (cartilage, bone marrow edema, erosion, or joint effusion) and graphed according to a colorimetric scale. Ligaments and menisci were also evaluated (n = 1,440 additional MRI measurements) but neither observer recorded damage to either structure in any knee at any time point. A total of n = 6,000 MRI measurements were recorded in this study.

### Harvest, dissection, and gross grading of knees

Bilateral hindlimb knees were harvested immediately after sacrifice by cutting through the thigh at the femoral mid-shaft and through the lower leg, distal to the tibial tuberosity, through the tibia and fibula. Skin was removed, exposing subcutaneous fat, and knees were submersed in 4% paraformaldehyde and stored at 4°C. Knees were examined during dissection for gross compromise of structure. The capsule was incised and the knee was opened through flexion, exposing the tibiofemoral joint plane. The capsular incision was carried up along the margins of the patellofemoral joint, the patella was reflected away from the trochlea, and the patellofemoral joint was exposed. Knees were graded immediately after dissection by one observer blinded to the knee treatment group. Extent of articular cartilage thinning and exposure of underlying subchondral bone was qualitatively determined for the entire intraarticular space by visual inspection as either normal, mild, moderate, or severe (numerically defined as 0, 1, 2, or 3, respectively). This constituted the global knee score. For compartment specific grading, a modified Osteoarthritis Research Society International (OARSI) scoring system was used[43] with the following adaptations. Gross articular cartilage was graded to a maximum of 4 at the medial and lateral tibial and femoral condyles and at the trochlea and patella (maximum score 24). Osteophytes were graded to a maximum of 3 at the medial and lateral tibial and femoral condyles and at the trochlea and patella (maximum score 18). Synovium was graded as suggested (maximum score 5)[43]. Similar to the method of depicting MRI grades by articular surface (above), gross grades were normalized to respective maximum scores and graphed according to % severity. Digital photographs were taken of the patellofemoral and tibiofemoral joints using a handheld digital camera.

### Euthanasia

Swine were deeply anesthetized and sacrificed in accordance with institutional policy for the humane sacrifice of animals. Intramuscular Telazol and xylazine was administered for anesthesia and euthanasia was performed by an intravenous injection of pentobarbital. Knees were harvested immediately after sacrifice as described above.

### Statistical analysis

To analyze the significance of different factors on lesion severity, a multivariate, ANOVA-type analysis using the function LMER in *R* was performed using time as repeated measures, drug as fixed effect, and animal as random effect. Inter-rater reliability assessed by calculating Cohen’s linear weighted kappa and percent agreement across ten different combinations of lesion category and joint region. Results were interpreted as previously described[44]. In *R*, kappa values were calculated using the function CKAP and percent agreement was determined using the function AGREE.

## Results

### Ultrasound-guided injection of MIA causes gross joint degeneration

**Fig 1:**
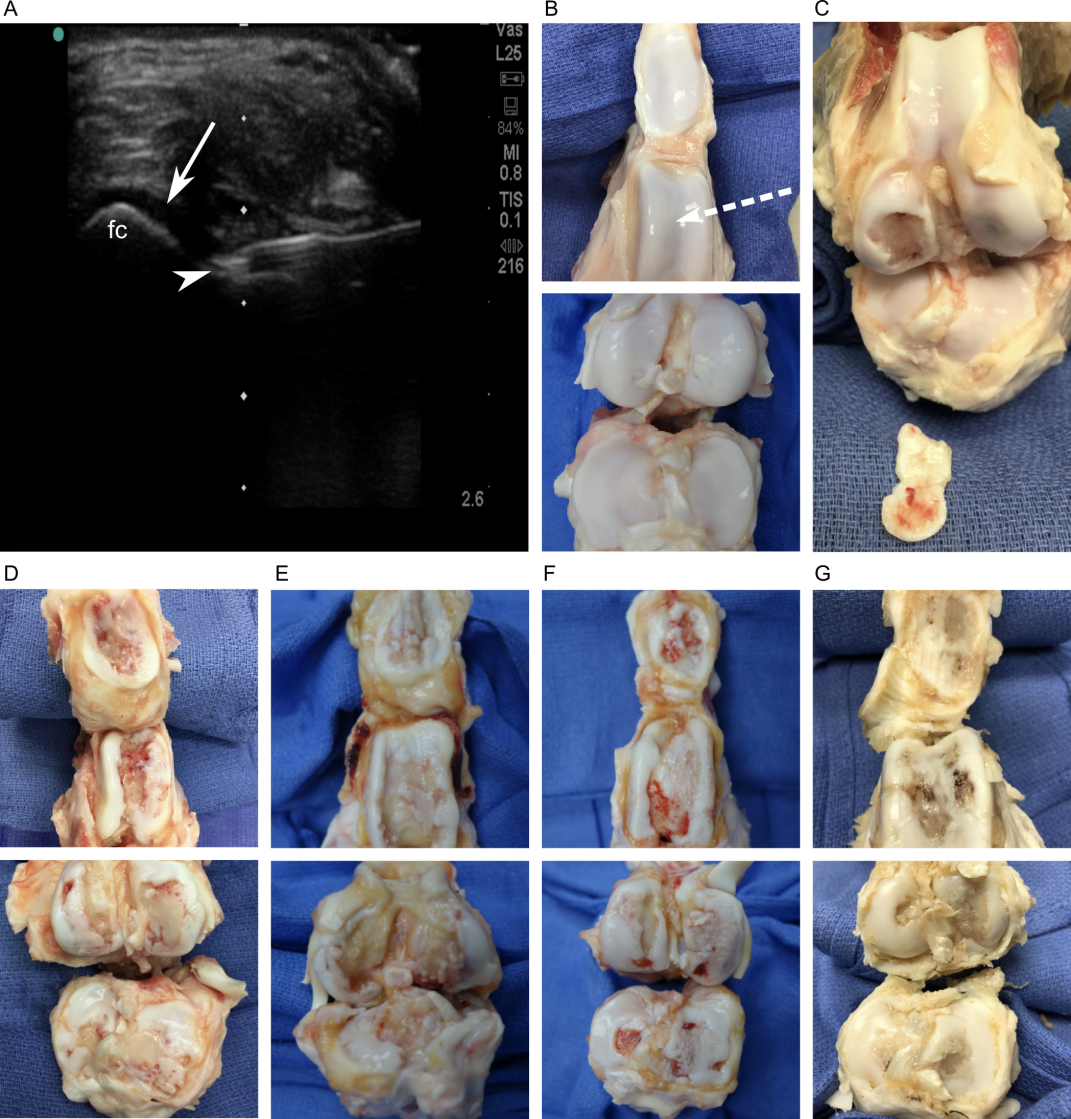
Gross assessment of MIA-, sham-, and non-injected knees. (A) Representative ultrasound image demonstrates application of the clinical intraarticular injection technique in swine. The needle tip (arrowhead) was guided in real time to the intraarticular space defined by the interface between hyperechoic cortical bone (femoral condyle, fc) and anechoic synovial fluid (arrow). (B) Representative image of a control knee (n = 29) showing intact articular cartilage of the patellofemoral (top) and tibiofemoral (bottom) articulations. The trajectory of the needle in panel A is illustrated (dotted arrow). (C) A non-injected knee illustrates traumatic, focal disruption of articular cartilage (n = 1) at the femoral condyle. The cartilage fragment shown outside the knee was found free-floating within the joint and tracing of its crisp borders showed it to match the focal area of exposed subchondral bone. This post-traumatic lesion was not factored into grading for this specimen. (D–G) Representative images of MIA-injected knees (n = 25) where articular cartilage destruction was unique compared to controls. (D) 40 mg MIA, n = 2. (E) 12 mg MIA, n = 2. (F) 4 mg MIA, n = 12. (G) 1.2 mg MIA, n = 9.

All control and experimental injections (n = 39) were carried out successfully, demonstrating that the clinical technique of ultrasound-guided joint injection was readily translated for use in swine (Fig 1A). Ultrasound allowed for near immediate identification of the patellofemoral articulation, which minimized the duration of anesthesia required to perform knee injections. Animals were sacrificed at various time points up to 35 weeks after injection (S2 Fig and S1 Table) and no animal developed signs of knee infection, skin breakdown, or leakage from the injection site throughout the study.

At the time of harvest, all n = 54 knees were dissected and graded. The extracapsular support system of tendons and ligaments were found to be intact and the articular capsule was not torn in any knee. Following dissection, the intraarticular surfaces of all knee joints appeared grossly sterile and without signs of suppuration. All intracapsular tendons, ligaments, and menisci were also intact. *Ex vivo* anterior and posterior drawer tests did not show ligamentous laxity in any specimen. Control knees did not exhibit gross cartilage destruction (Fig 1B) except for n = 1 knee. This specimen was the contralateral, non-injected member of a 4 mg MIA knee found to contain an intraarticular, free-floating piece of cartilage that was a physical match to a single area of exposed subchondral bone (Fig 1C). This lesion was focal and well circumscribed, contrasting the roughened, creeping erosion of MIA (Fig 1D–1G). A review of veterinary records showed that this animal developed a spontaneous limp in the same hindlimb that resolved after three days. Given the clinical history and postmortem morphology, this lesion was considered to be of traumatic etiology, i.e., not due to MIA, and was excluded from gross grading of that specimen.

MIA-injected knees exhibited gross articular cartilage thinning and erosion, exposure of underlying subchondral bone, and eburnation (Fig 1D–1G). These findings were unique to MIA-injected knees and overall severity of cartilage destruction and exposure of subchondral bone ranged from mild to severe. Control knees (sham: n = 14 and non-injected: n = 15) demonstrated intact articular cartilage at all sacrifice time points (S2 Fig). Knees injected with 40 mg MIA (n = 2) showed severe cartilage destruction by week 17 and 12 mg knees (n = 2) showed moderate or severe damage at week 18 or 19, respectively. The 4 mg group (n = 12) comprised an equal representation of grades: severe damage or mild and moderate combined (n = 6 each). The 1.2 mg group demonstrated a predominance of moderate cartilage damage (n = 6). Mild damage was exclusive to the 1.2 and 4 mg dose groups. Detailed grading of gross pathology for each articular surface and the surrounding synovium are depicted for MIA-injected knees (S3 Fig).

### Structural characterization of MIA-induced joint destruction by MRI

**Fig 2:**
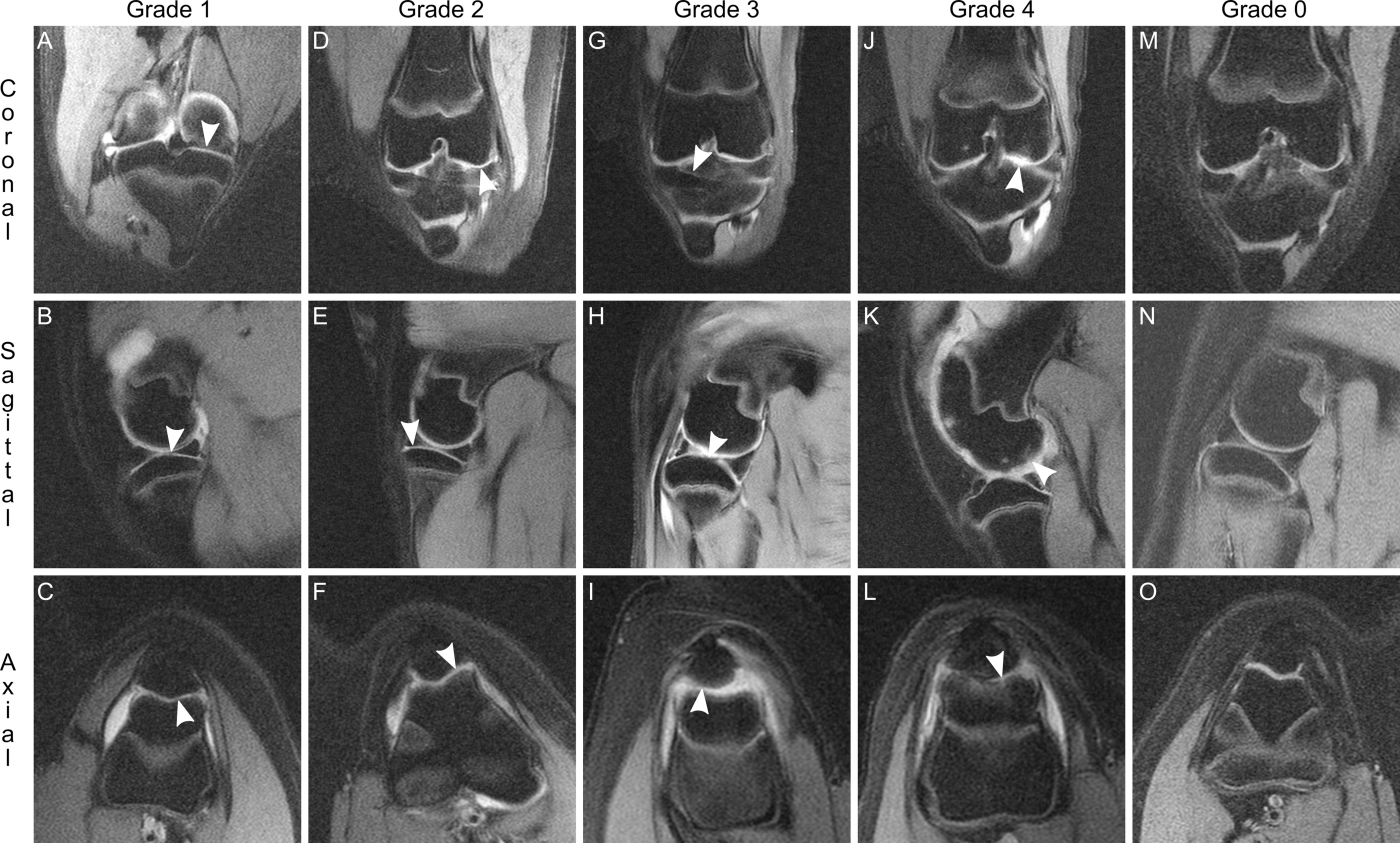
Graded destruction of articular cartilage. Articular cartilage was seen on multiplanar MR images as a band hyperintense (white) signal between the intraarticular space and subchondral bone. The thickness of this band was compared throughout its length and the quality and degree of its thinning was used to grade each cartilage lesion. Grade 1 damage overlying the tibial plateau (A and B) and patella (C), as indicated by nearly normal appearance with superficial indentation (arrowheads), here from one 4 mg knee at week 8. Grade 2 damage overlying the femoral condyle (D), tibial plateau (E), and trochlea (F), as indicated by cartilage thinning of less than 50% (arrowheads), here from two 4 mg knees at weeks 8 and 9. Grade 3 damage overlying the tibial plateau (G and H) and patella (I), as indicated by loss of cartilage of at least 50% and up to subchondral bone but not deeper (arrowheads), here from two 4 mg knees at weeks 6 and 8. Grade 4 damage overlying the femoral condyle (J and K) and trochlea (L), as indicated by complete loss of cartilage with associated underlying damage to subchondral bone (arrowheads), here from one 4 mg knee at week 12. Representative Grade 0 images from one non-injected knee contralateral to a 4 mg knee at week 12 (M, N, O) demonstrate full thickness cartilage throughout all articular surfaces. Images are representative of n = 587 cartilage lesions identified. Coronal views: top row. Sagittal views: middle row. Axial views: bottom row.

**Fig 3:**
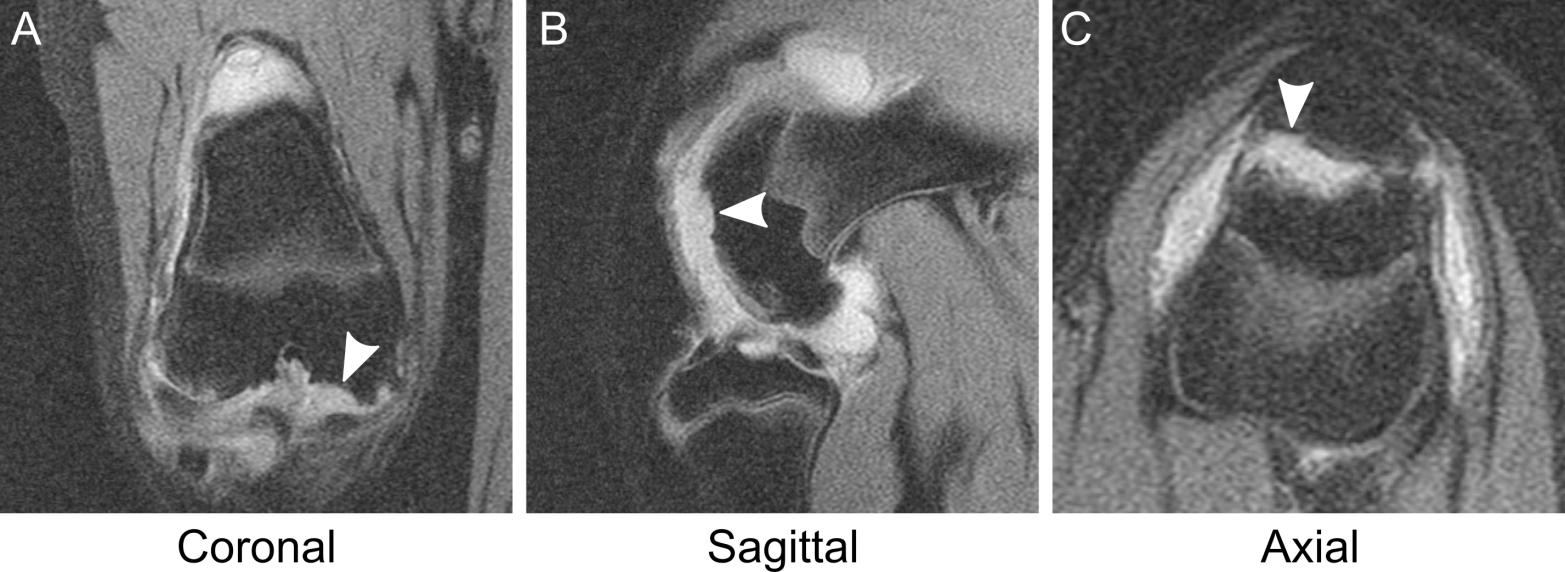
Subchondral bone erosion. (A) Coronal view and (B) sagittal view of an erosion in the femoral condyle (arrowheads). The lesion is a discrete, scalloped indentation in the subchondral bone associated with the absence of overlying cartilage. (C) Axial view of another erosion located in the patellar subchondral bone (arrowhead), here from one 4 mg knee at week 12. The overlying articular cartilage is completely missing. Images are representative of n = 292 erosions identified.

**Fig 4:**
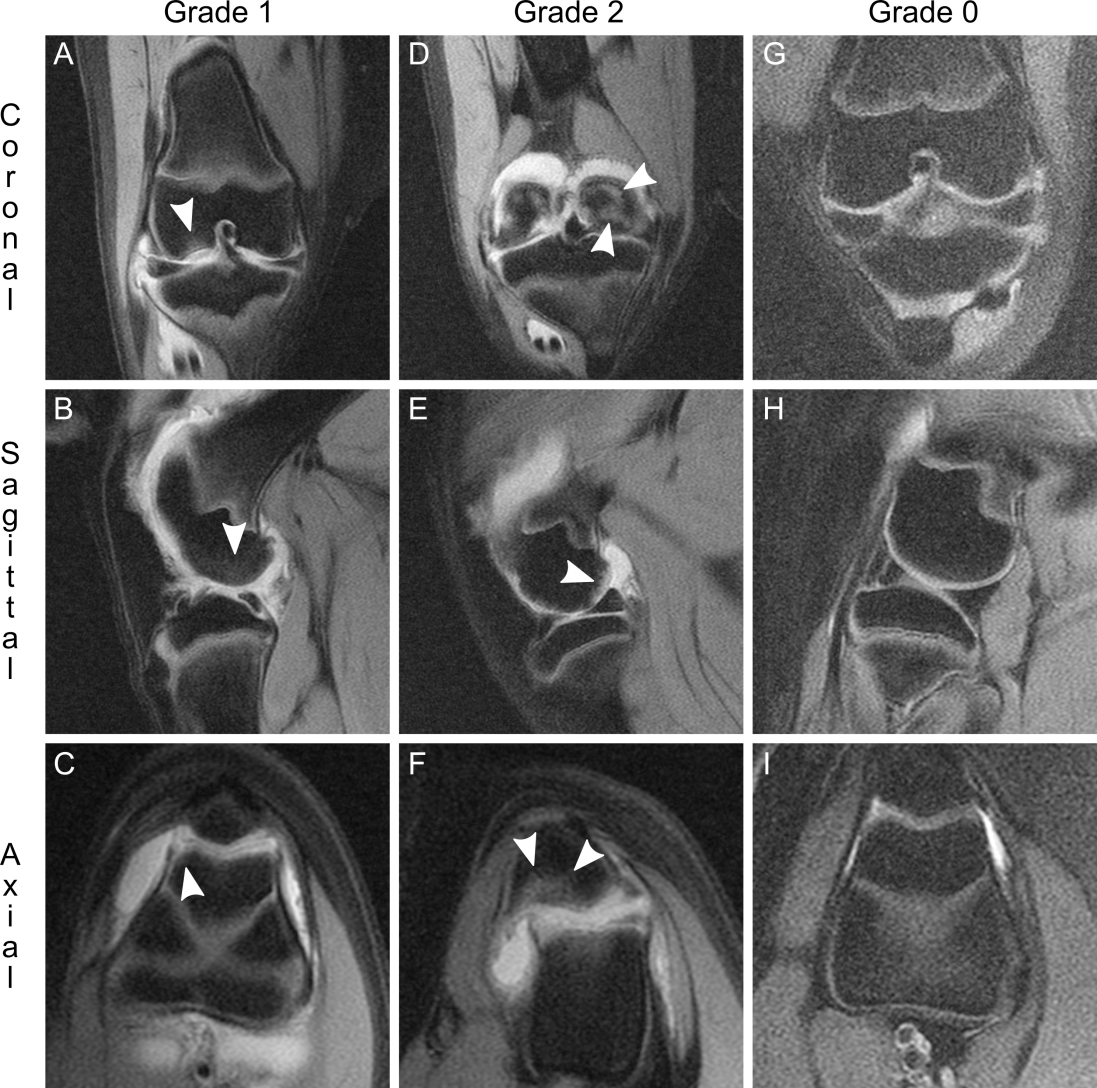
Bone marrow edema. Edema is seen in subchondral bone as a hyperintense focus extending from the cartilage-bone interface into underlying bone. The deepest, leading edge of edematous signal is serpiginous and irregular. Grade 1 bone marrow edema is evidenced by relatively small hyperintense foci (arrowheads), extending here into subjacent subchondral bone of the femoral condyle (A and B) and the trochlea (C), here from two 4 mg knees at week 7. Grade 2 bone marrow edema (arrowheads) is relatively moderate in size, shown at the posterior aspect of the femoral condyle (D and E) and within the patella (F), here from one 4 mg knee at week 10. Grade 3 bone marrow edema was not found in any knee throughout the study. Representative Grade 0 images from one non-injected knee contralateral to a 4 mg knee at week 12 (G, H, I) demonstrate absence of subchondral hyperintensity. Images are representative of bone marrow edema identified in n = 412 images. Coronal views: top row. Sagittal views: middle row. Axial views: bottom row.

**Fig 5:**
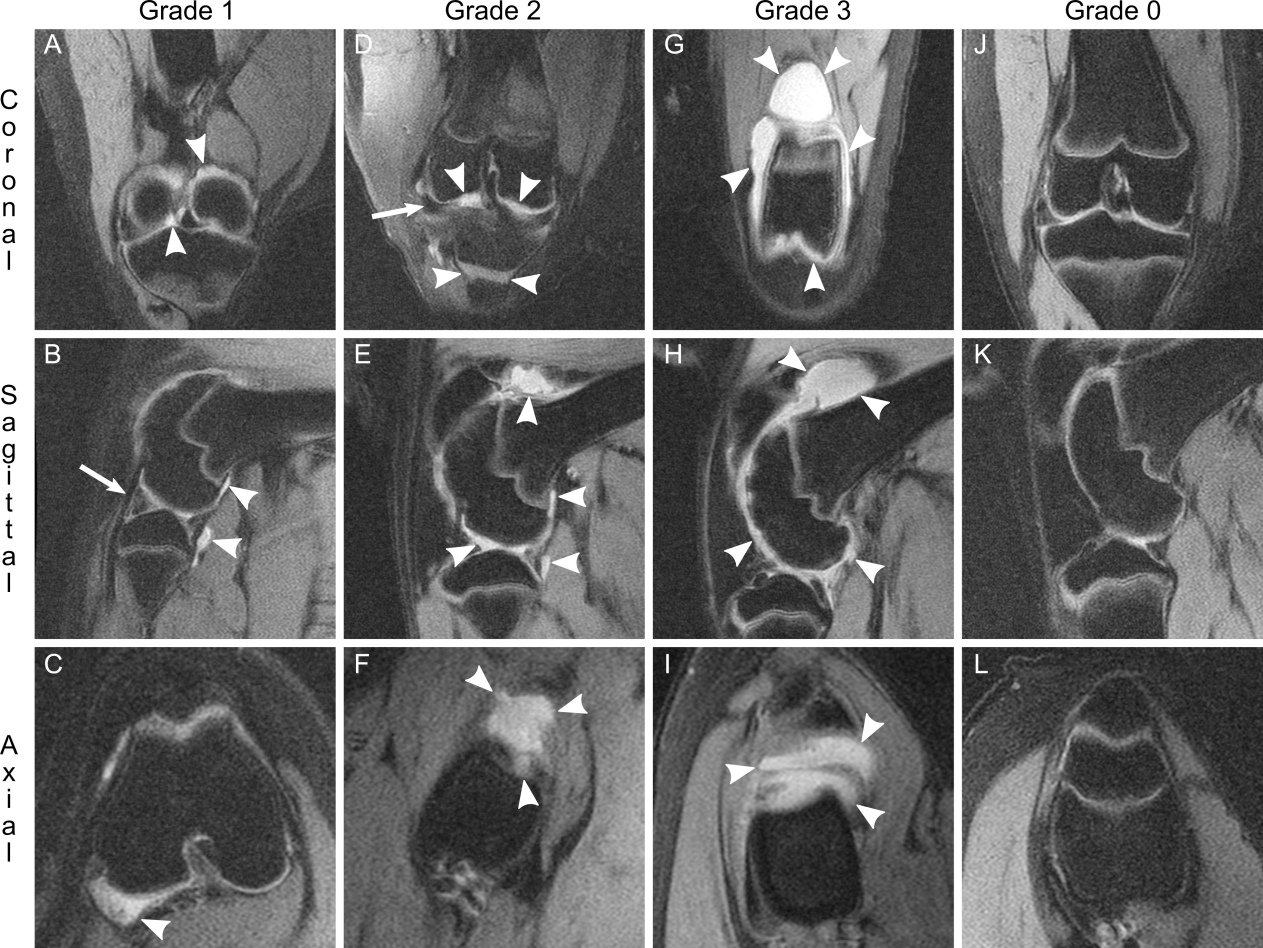
Intraarticular joint effusion. Joint effusions were observed as hyperintense fluid collections (arrowheads) within the joint space of MIA-injected knees. Control knees never demonstrated an effusion. Grade 1 effusions were small and observed as focal fluid collections present along the margins of the femoral condyles (A and B, upper arrowhead and C) and the tibial condyle (B, lower arrowhead), here from one 4 mg knee at week 12. Grade 1 lesions did not appear to distend the intraarticular capsule. Grade 2 effusions were of moderate size and formed multiple, discrete collections in a single joint, for instance, along the tibia plateau (D), femoral condyle, and femoral shaft (E), here from one 4 mg knee at week 8. Grade 2 effusions distended the intraarticular capsule in several locations, including at the suprapatellar space (E, upper arrowhead and F). Grade 3 lesions formed circumferential collections around intraarticular bone (G and H) and displaced extraarticular soft tissue (I), here from one 4 mg knee at week 12. Examples of an intact intraarticular tendon (B, arrow) and intact meniscus (D, arrow) are shown for reference. Representative Grade 0 images from one non-injected knee contralateral to a 4 mg knee at week 12 (J, K, L) demonstrate absence of intraarticular hyperintense fluid. Images are representative of n = 97 joint effusions identified. Coronal views: top row. Sagittal views: middle row. Axial views: bottom row.

3T MRI provided the spatial resolution required for characterizing structural degradation that occurred in MIA-injected knees. Image slices in three dimensions (coronal, sagittal, and axial) were used by two observers to grade lesions (S1 Fig). Three-dimensional evaluation was critical for defining the shape and size of individual lesions and to confirm or deny any lesion suspected to exist in a single plane. Two observers identified n = 587 cartilage lesions (Fig 2), n = 704 lesions in subchondral bone (Fig 3 and Fig 4), and n = 97 joint effusions (Fig 5).

On fat-saturated, proton density sequences, articular cartilage was identified as a hyperintense band, superficial to subchondral bone. Compromise of articular cartilage presented as relative thinning along its course. Individual cartilage lesions demonstrated an area of maximum thinning which determined severity. Superficial indentations appearing “nearly normal” were assigned grade 1 (Fig 2A–2C). Cartilage thinning less than 50% (Fig 2D–2F) or from 50% up to subchondral bone (Fig 2G–2I) was graded 2 or 3, respectively. Lesions that extended completely through cartilage and were associated with damage to underlying subchondral bone received a grade of 4 (Fig 2J–2L). Grade 4 lesions were invariably associated with a discrete focus of subchondral bone loss. These lesions signified the presence of an erosion (Fig 3). The inverse was also true: no erosion was observed in isolation without overlying cartilage destruction of grade 4.

Bone marrow edema was observed on MRI as hyperintense foci that extended from the cartilage-bone interface (subchondral plate) and into adjacent subchondral bone (Fig 4). The leading edge of each lesion was identified by its irregular, serpiginous margin demonstrated in all three imaging planes. Identification of edema in this way allowed for differentiation from the second type of subchondral lesion characterized; an erosion (Fig 3). Severity of bone marrow edema was graded according to lesion size, specifically small (Fig 4A–4C) or moderate (Fig 4D–4F). A third level edema severity was included in the grading scheme but was never observed.

Joint effusions were, visually, the most striking lesion. Effusions were demarcated as intraarticular collections of hyperintense fluid (Fig 5). Grade 1 effusions were small and observed to spread along the margins of intraarticular bone as smooth-bordered, hyperintense collections (Fig 5A–5C), eventually distending the capsular edges in cases of grade 2 effusion (Fig 5D–5F). Grade 3 effusions displaced adjacent, extraarticular soft tissue (Fig 5G–5I). All effusions were found to be contained within the intraarticular space and did not breach the joint capsule. A capsular breach would have been identified by the presence hyperintense signal in adjacent soft tissue, signifying extravasation of synovial fluid. Other intraarticular structures, the cruciate ligaments, tendons, and menisci, appeared intact and appropriately aligned at all time points (Fig 5B and 5D). This observation correlated with the finding that these structures were also intact at sacrifice.

### Reliability of MRI grading

Inter-rater reliability for MRI grading performed by two radiologists blinded to the injectate was interpreted as suggested in the literature[44] and agreement met or exceeded that reported in other image-based OA models[45–47]. Grading of cartilage damage at the lateral tibiofemoral and patellofemoral regions, bone marrow edema at the lateral tibiofemoral, medial tibiofemoral, and patellofemoral regions, and subchondral erosions at the lateral tibiofemoral and patellofemoral regions was “almost perfect” or “strong” (Table 1). Grading of cartilage damage at the medial tibiofemoral, subchondral erosion at the medial tibiofemoral region, and effusions was “moderate” or “weak” (Table 1).

**Table 1:** Inter-rater reliability of MRI grading.

Lesion category	Knee region	Cohen’s linear weighted kappa (95% confidence interval)^a^	Percent agreement
Cartilage damage	Lateral tibiofemoral	0.81 (0.76–0.86)	77.9
	Medial tibiofemoral	0.78 (0.73–0.83)	72.5
	Patellofemoral	0.89 (0.85–0.94)	89.6
Bone marrow edema	Lateral tibiofemoral	0.82 (0.76–0.88)	89.2
	Medial tibiofemoral	0.81 (0.74–0.88)	90.4
	Patellofemoral	0.82 (0.75–0.88)	90.0
Subchondral erosion	Lateral tibiofemoral	0.81 (0.71–0.91)	94.6
	Medial tibiofemoral	0.56 (0.41–0.69)	87.1
	Patellofemoral	0.96 (0.92–1.00)	98.3
Effusion	Total intraarticular space	0.77 (0.69–0.85)	76.7

^a^Interpretation of agreement based on Cohen’s kappa values was performed as suggested in the literature[44]. Almost perfect: above 0.90, strong: 0.80–0.90, moderate: 0.60–0.79, weak: 0.40–0.59, minimal: 0.21–0.39, none: 0–0.20.

### MRI accurately depicts structural changes seen on gross pathology

Control and MIA-injected knees harvested one week after final imaging were used to assess the accuracy of MRI in depicting gross structure (n = 14). Structural damage to cartilage identified on MRI (Panels A–F in S4 Fig, top panels) were identified at corresponding locations in harvested knees (Panels A–F in S4 Fig, bottom panels). No significant difference was found for the severity of cartilage damage when graded on MRI or at the time of harvest (Panel G in S4 Fig). This result suggests that MRI is an accurate method for *in vivo* evaluation of structural damage to cartilage after MIA injection.

### Characterization of MIA-induced OA by MRI

**Fig 6:**
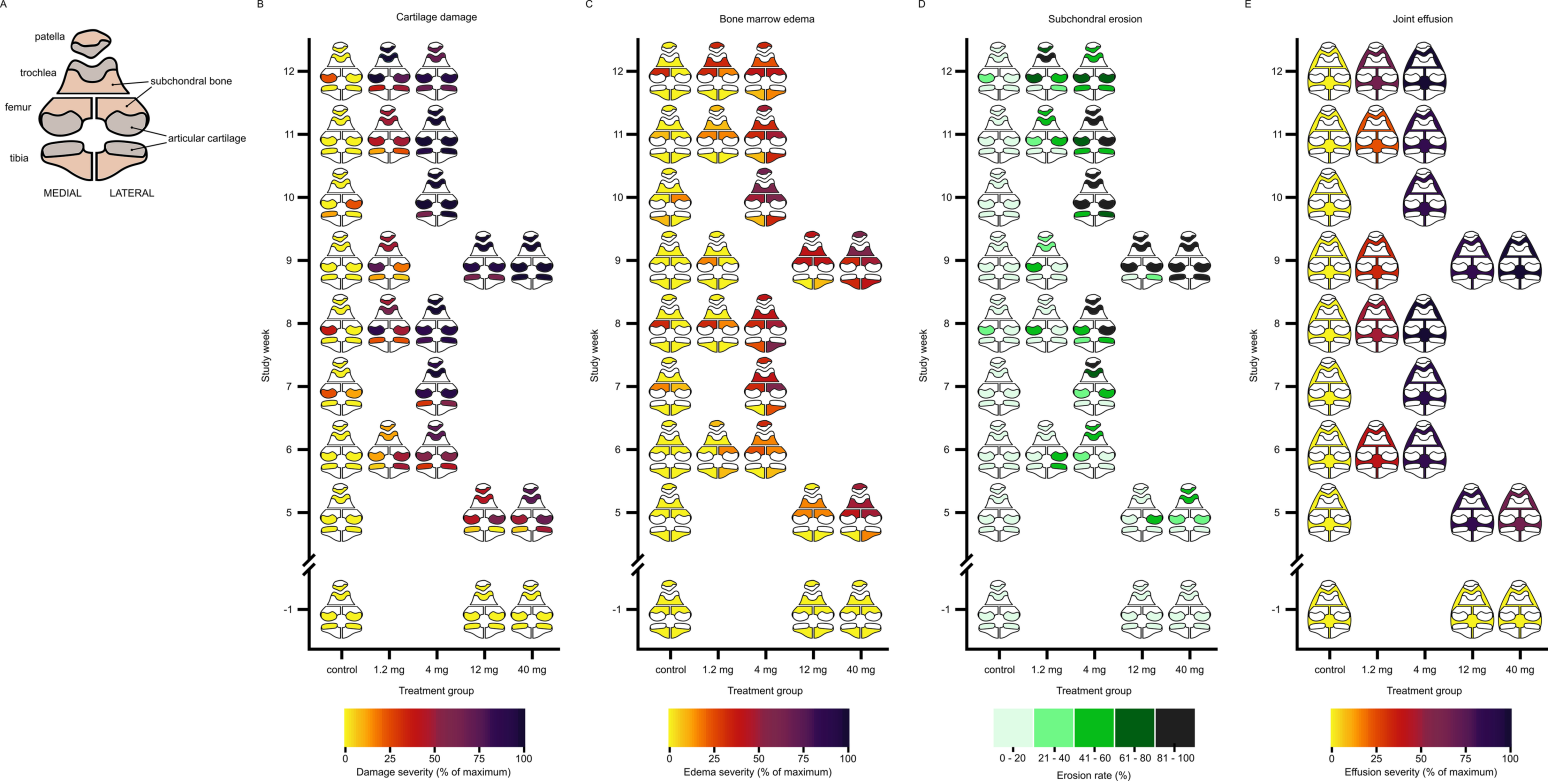
Progression of structural joint damage quantified by serial MRI. (A) Idealized knee depicts the anatomical relationship between paired articular surfaces and bones comprising the knee joint in humans and swine. A total of n = 4,560 MRI measurements are summarized as anatomical heat maps. The coloring of articular surfaces, subchondral bone, and the intraarticular space relates lesion severity and location shown in the remaining panels. Lesion severity is indicated by different colors. Detailed methods for computing numerical grades and converting grades into color is described in the methods section. Minimal or no damage was seen at the outset of the study, reflecting healthy knees (lighter colors) for cartilage damage (B), bone marrow edema (C), subchondral erosion (D), and joint effusion (E). Lesion severity increased in each category with time and dose. Ligaments and menisci were also evaluated (n = 1,440 MRI measurements) but neither observer recorded damage to either structure in any knee at any time point.

Lesion severity at different joint locations (Fig 6A) observed in MIA-injected knees was progressive for all categories, including cartilage damage (Fig 6B), bone marrow edema (Fig 6C), erosions (Fig 6D), and effusions (Fig 6E). Onset of disease was relatively more rapid for higher dose groups. Within individual weeks, lesion severity increased with dose but by the final time point, all groups had progressed to the same level of joint compromise. A multivariate, ANOVA-type analysis showed that time was a highly significant factor for lesion severity (*P* = 2.29x10^-10^). At the group sizes tested, dose level was not significant when the control group was excluded from the analysis. If controls were included, i.e., a binary comparison for exposure to MIA, dose became a significant factor.

Cartilage damage reached maximum severity for the 4, 12, and 40 mg groups at the majority of articular surfaces by weeks 8 to 9, damage occurred at the femur and patellofemoral joint first, followed by the tibia (Fig 6B). During week 12, the 1.2 mg group achieved near maximum cartilage damage restricted to the femur and patellofemoral joint. Leading up to the final week, the 1.2 mg group demonstrated a period of medium severity in the range of 40% to 75% of maximum during weeks 8 to 11. A similar period of moderate damage was not observed in the other dose groups. The severity of bone marrow edema observed in MIA-injected knees exhibited similar progression compared with cartilage but never reached the same peak severity (Fig 6C). The greatest edema was seen at the trochlea, patella, and femur in the 40 and 4 mg dose groups during weeks 9 and 10, respectively. The 1.2 mg dose group demonstrated its greatest edema at the medial femur, trochlea, and patella, achieving 50% maximum severity by weeks 11 and 12. Similar to cartilage, bone marrow edema severity increased with dose during individual weeks. The rate of erosion reached peak levels at all surfaces by week 9 for the 40 mg group (Fig 6D). Erosions progressed more slowly and affected fewer articular surfaces in the remaining dose groups. The majority of erosions in these groups were focused at the femur, trochlea, and patella, with a minority occurring at the tibia. The occurrence of joint effusions preceded other categories (Fig 6E). All MIA-injected knees demonstrated rapid onset of effusions to 100% maximum severity except for the 1.2 mg group, which demonstrated a period of stability near 50% maximum severity from weeks 6 to 11 before peaking at 75% maximum severity.

MRI of control knees revealed evidence of spontaneous joint disease. Control lesions were infrequent and were transient when they did occur. Spontaneous cartilage damage and bone marrow edema in control knees did not exceed 40% maximum severity (Fig 6B and 6C) or 40% maximum erosion rate (Fig 6D). Joint effusions never occurred in control knees and were therefore specific to MIA-injected knees (Fig 6E). The majority of spontaneous lesions occurred together, i.e., lesions arose and resolved at the same time and location. Interestingly, control knees never exhibited patellofemoral degradation. Pathology at that site was therefore specific to MIA-injected knees and in the majority of cases, the trochlea and patella progressed together more often than any other articular pair (Fig 6A). This observation suggests that the biochemical effect of MIA was greatest near the intraarticular site of injection, which was the space between the patella and trochlea.

## Discussion

A new large animal model of MIA-induced OA was developed with comprehensive characterization by clinical quality 3T MRI. In swine, MIA caused the principal findings of gross joint degradation reported in other animal models of OA[17,48–55], including cartilage loss, exposure of subchondral bone, and eburnation. The observation that OA presented consistently in all experimental knees underscores the technical precision achieved by ultrasound guidance in delivering MIA to the intraarticular space. Clinical MRI was an accurate method for *in vivo* characterization of gross structural change in the porcine knee. We tailored the sequence acquisition parameters to produce high resolution, three-dimensional images of joint-specific structures, supporting reliability of grading between two blinded radiologists. Quantitation of MRI-pathology was achieved with a well-defined grading system adopted from clinical use that was shown to yield reproducible results with high degree of inter-rater agreement meeting or exceeding reference standards reported in other image-based OA models[45–47].

The most ambitious goal in modeling of OA would be to simulate human joint disease in a manner that facilitates preclinical testing of novel drugs or other disease modifying interventions. The ideal model would demonstrate rapid onset of OA, titratability of peak severity, and prolonged stability of the diseased phenotype. Titratability of peak severity would allow the model to be used for testing symptom-targeted drugs in mild or moderate OA and disease modifying interventions aimed at slowing or reversing progression to severe OA. Rapid onset and subsequent prolonged stability of the disease phenotype would be ideal features of a model to permit repeat testing for treatment effects within cohorts. Variation between animals should be small compared to measured outcome parameters.

In the model reported here we demonstrated methods of OA induction and MRI analysis that were rigorous and reliable. That approach allowed us to critically assess the characteristics of the model, including that we discovered limitations not due to technical parameters but caused by the natural course of MIA-induced OA in swine. We expected that MIA dose would be the main determinant of OA severity because others have reported dose-dependent outcomes in small animals[16,53,54,56,57]. We therefore tested a wide range of MIA doses–four dose levels spaced at 3.3 fold increases–in order to discern the useful concentrations for titrating model severity. In the majority of damage categories, a trend towards dose dependency was found but with substantial overlap in outcomes between adjacent dose levels. Attempts to explain the variation led us to closely look at the evolution of OA severity over time.

A main insight was that the dominant determinant of OA severity was time elapsed since MIA administration. Progression of OA severity was relentless for the entire study period resulting in severe joint destruction by 12 weeks for animals from any intermediate or high dose group. OA progression did not appear to come to a halt or to slow at any time during the experiment for any of the MIA doses; thus a plateau of stable OA was not found. Minor variation in the rate of progression, i.e. in the speed with which the natural evolution of OA occurred in individual animals, likely accounted for a substantial portion of the inter-individual (between-subject) variability recorded. Essentially, any difference in OA severity observed at a given time point between replicate animals in any of the dosing cohorts was no more than the difference observed systematically for all animals when compared two or three weeks later.

We were interested in understanding the relative contribution of time and MIA dose quantitatively. Towards that, we estimated the effect of time on OA severity relative to the effect of dose with the help of the ANOVA-type multivariate model employed for analysis. We found that two additional weeks of disease progression (a short time frame in the scope of the experiment) could account for the same difference in OA severity as a 10-fold increase in the dose of MIA (a large jump in the scope of the experiment). Therefore, while MIA dose was significant on three or four subscales of OA severity, it had a smaller effect size than time across all conditions tested.

In summary, we established a new model of experimental OA with image-based assessment of severity by intraarticular MIA in swine that recapitulates findings of MIA OA in small animals and key characteristics of human OA[5,12,13,15]. We sought the use of swine in order to overcome the size discrepancy between rodent and human joints, and to provide researchers with a critical option of species matched to emerging laboratory and institutional preferences, specifically, employing a use animal (swine), rather than the previous emphasis on a companion animal (dogs). Multiple joint regions were quantified in swine *in vivo* using serial MRI and the adapted clinical grading scheme was a reliable method to assess lesion severity. Furthermore, this model supports the field by adding extensive use data on 3T MRI imaging that reflects equipment in current use for the diagnosis of complex joint diseases in the clinic. The underlying mechanism of MIA joint injury, with widespread cell death and rapid destructive change, differs from spontaneous or post-traumatic OA. This should be acknowledged in any study using the model. Shortcomings of such as this appear to be inherent to model creation, i.e., the need to produce pathology reproducibly and within a practical time-frame seems to necessitate the use of pathology-inducing procedures that might mirror the naturally occurring disease state only incompletely. In support of the MIA model in swine, it could be considered that it retains face validity in that its inter-individual heterogeneity and progression over time are resembling human OA. Furthermore, the model makes the human-scaled anatomical characterization possible. The serialized, in vivo detail offered by 3T MRI in the current study may improve translation of extensive, pre-existing rodent MIA OA data previously restricted due to size discrepancy alone.

## Supporting information

S1 Fig Animal positioning and acquisition of hindlimb knee MR images in three dimensions. (A) After induction of anesthesia and intubation, swine were positioned in the right lateral decubitus position with the left knee up for imaging the left knee (as shown). To image the right knee, swine were positioned in the left lateral decubitus position with the right knee up. Animals entered the MRI bore rump (R) first. Liberal padding (P) was used to ensure the animal remained comfortable, free from nerve compression or skin irritation, and with the knee in a partially extended position. (B) After appropriate padding was placed, a wide fastening strap (S) with padded undersurface was gently secured. This guaranteed that the limbs and their positioning would be maintained as the MRI table (T) was moved into and out of the bore. (C) Representative midsagittal MR scout image of a swine knee. Dotted blue lines indicate individual image slices that were acquired in the coronal plane. Coronal slices were acquired parallel to the plane of the patellofemoral joint (arrows). (D) Representative midsagittal MRI scout image of a swine knee. Dotted blue lines indicate individual image slices that were acquired in the axial plan. Axial slices were acquired perpendicular to the patellofemoral joint (arrows). The white squares indicate the shaft of the femur, which is the proximal boney member of the tibiofemoral joint, and the white circle indicates the shaft of the tibia, which is the distal boney member of the tibiofemoral joint. The white square and circle are shown in (A) for orientation purposes.
(TIF)

S2 Fig Timeline of study events. Time points for MRI and sacrifice are depicted for each animal. Week 0 corresponds to the time of MIA injection. All points for a given animal are colored to indicate the dose of MIA (blue: 0 mg, red: 1.2 mg, green: 4 mg, purple: 12 mg, orange: 40 mg).
(TIF)

S3 Fig Grading of gross pathology. (A) Bilateral hindlimb knees were examined from n = 27 animals and cartilage damage was analyzed according to a global qualitative grading system (intact, mild, moderate, or severe disruption) by a blinded observer. (B) Stylized anatomical models of MIA-injected knees are shown outlining the compartment specific grades for cartilage and osteophytes and a global knee grade for synovial pathology. Each model is numbered to indicate the corresponding data point in A. (C) Legend for the stylized model illustrates the coding of gross data in B. Each point in A represents one knee (n = 54). Injected knees are represented by closed symbols and non-injected knees are represented by open symbols. Sham knees received a 2 ml injection of PBS. The control group consisted of both sham and non-injected knees.
(TIF)

S4 Fig Close correlation of structural changes identified *in vivo* and at sacrifice. Representative MR images (A–F, top panels) obtained one week prior to gross images (A–F, bottom panels). A total of n = 14 knees were found to have been harvested one week after final MR imaging (bilateral hindlimb knees from 4 mg MIA animals, namely B773, P20, P21, P22, P23, P24, P25). Thinning of articular cartilage overlying the tibial plateau was identified, ranging from partial (arrows, A and B) to complete (asterisk, C). (D) An interlocking patellofemoral erosion revealed a complete grade 4 lesion of patellar cartilage with subchondral erosion (brackets) but not at adjacent trochlea cartilage (arrows, D). (E) Midsagittal invagination of the femoral groove illustrated the effect of friction on thinning damaged cartilage (arrows). (F) MRI revealed isolated fissures and undulating rents in patellar cartilage that were confirmed upon gross inspection (arrows). (G) No significant difference existed between the MRI and gross grades of cartilage damage (*P* = 0.34). Cartilage grades from all six surfaces of each knee were totaled and divided by the numerical maximum score obtainable (3) to give a normalized grade (percent of maximum). Values were expressed as the mean, ± confidence interval. Statistical significance (*P* < 0.05) was determined using the paired Student’s *t*-test (two-tailed) between the two modes of evaluation.
(TIF)

S1 Table Summary of swine characteristics used in this study. (DOCX)
