# Supplementary figures and images for: Clinical magnetic resonance-enabled characterization of mono-iodoacetate-induced osteoarthritis in a large animal species
Source: PLoS One. 2018 Aug 3;13(8):e0201673. doi: 10.1371/journal.pone.0201673 (PMC6075758; doi:10.1371/journal.pone.0201673)

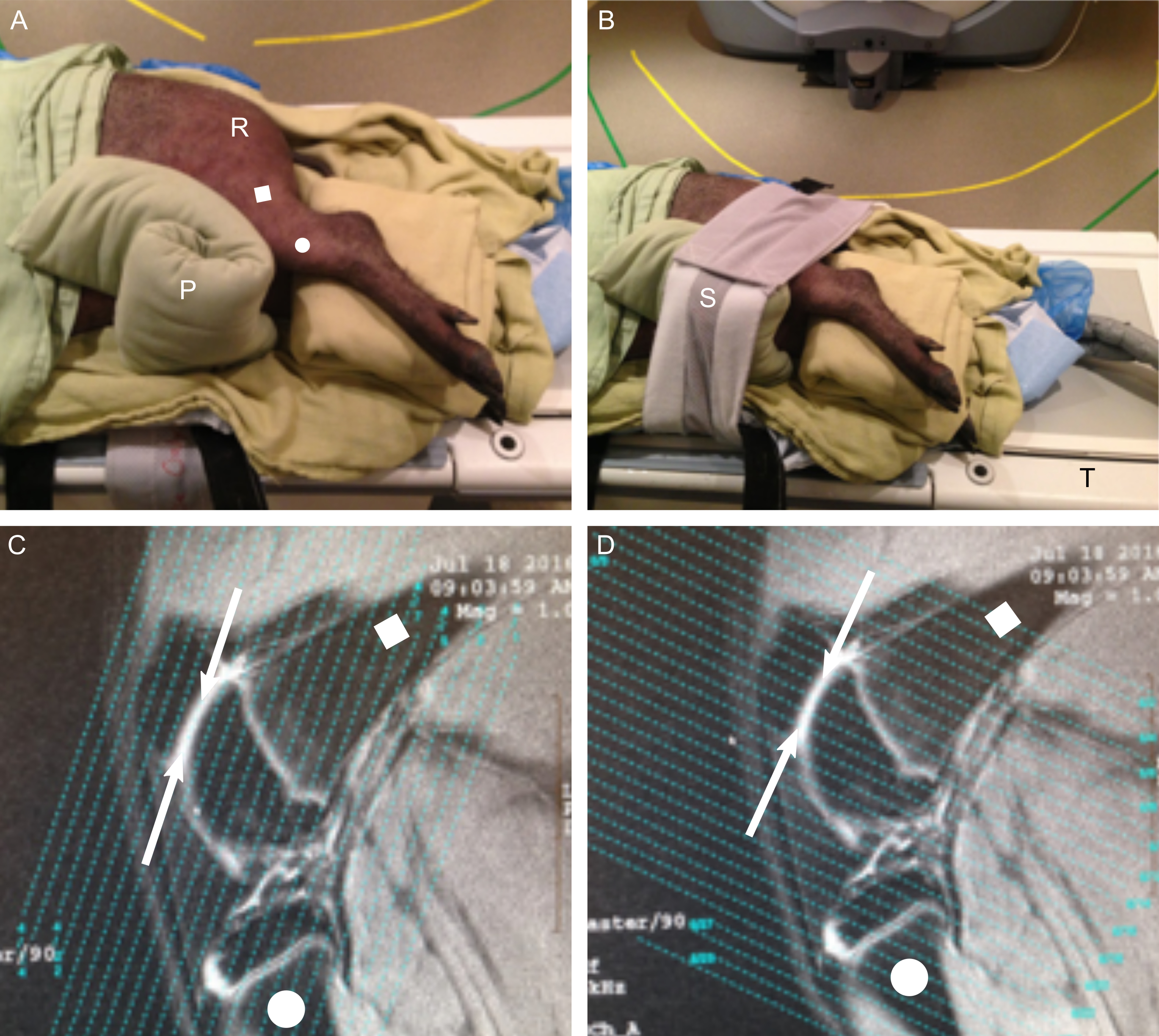

Supplement: S1 Fig — (A) After induction of anesthesia and intubation, swine were positioned in the right lateral decubitus position with the left knee up for imaging the left knee (as shown). To image the right knee, swine were positioned in the left lateral decubitus position with the right knee up. Animals entered the MRI bore rump (R) first. Liberal padding (P) was used to ensure the animal remained comfortable, free from nerve compression or skin irritation, and with the knee in a partially extended position. (B) After appropriate padding was placed, a wide fastening strap (S) with padded undersurface was gently secured. This guaranteed that the limbs and their positioning would be maintained as the MRI table (T) was moved into and out of the bore. (C) Representative midsagittal MR scout image of a swine knee. Dotted blue lines indicate individual image slices that were acquired in the coronal plane. Coronal slices were acquired parallel to the plane of the patellofemoral joint (arrows). (D) Representative midsagittal MRI scout image of a swine knee. Dotted blue lines indicate individual image slices that were acquired in the axial plan. Axial slices were acquired perpendicular to the patellofemoral joint (arrows). The white squares indicate the shaft of the femur, which is the proximal boney member of the tibiofemoral joint, and the white circle indicates the shaft of the tibia, which is the distal boney member of the tibiofemoral joint. The white square and circle are shown in (A) for orientation purposes. (TIF) [file pone.0201673.s001.tif]

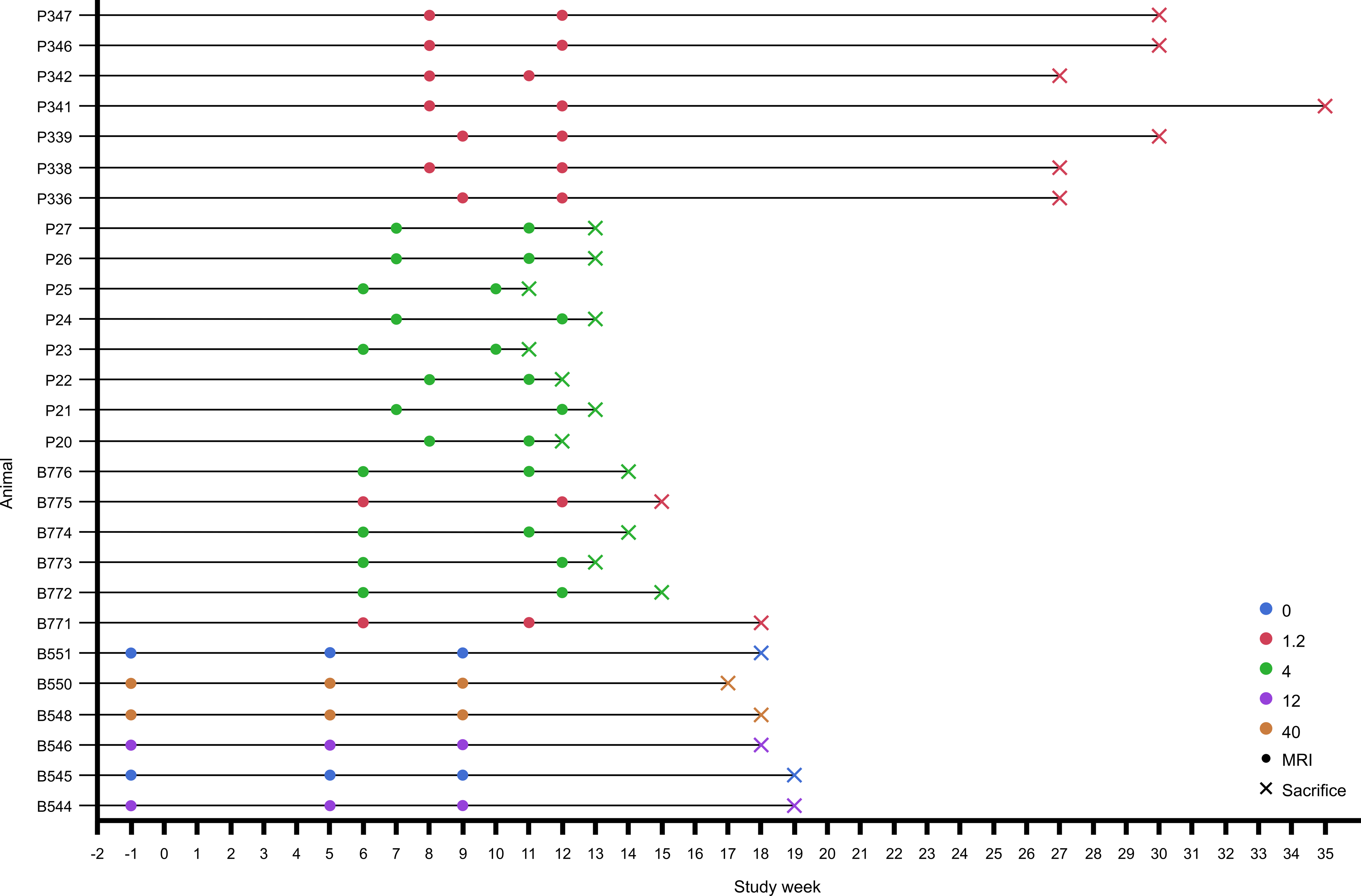

Supplement: S2 Fig — Time points for MRI and sacrifice are depicted for each animal. Week 0 corresponds to the time of MIA injection. All points for a given animal are colored to indicate the dose of MIA (blue: 0 mg, red: 1.2 mg, green: 4 mg, purple: 12 mg, orange: 40 mg). (TIF) [file pone.0201673.s002.tif]

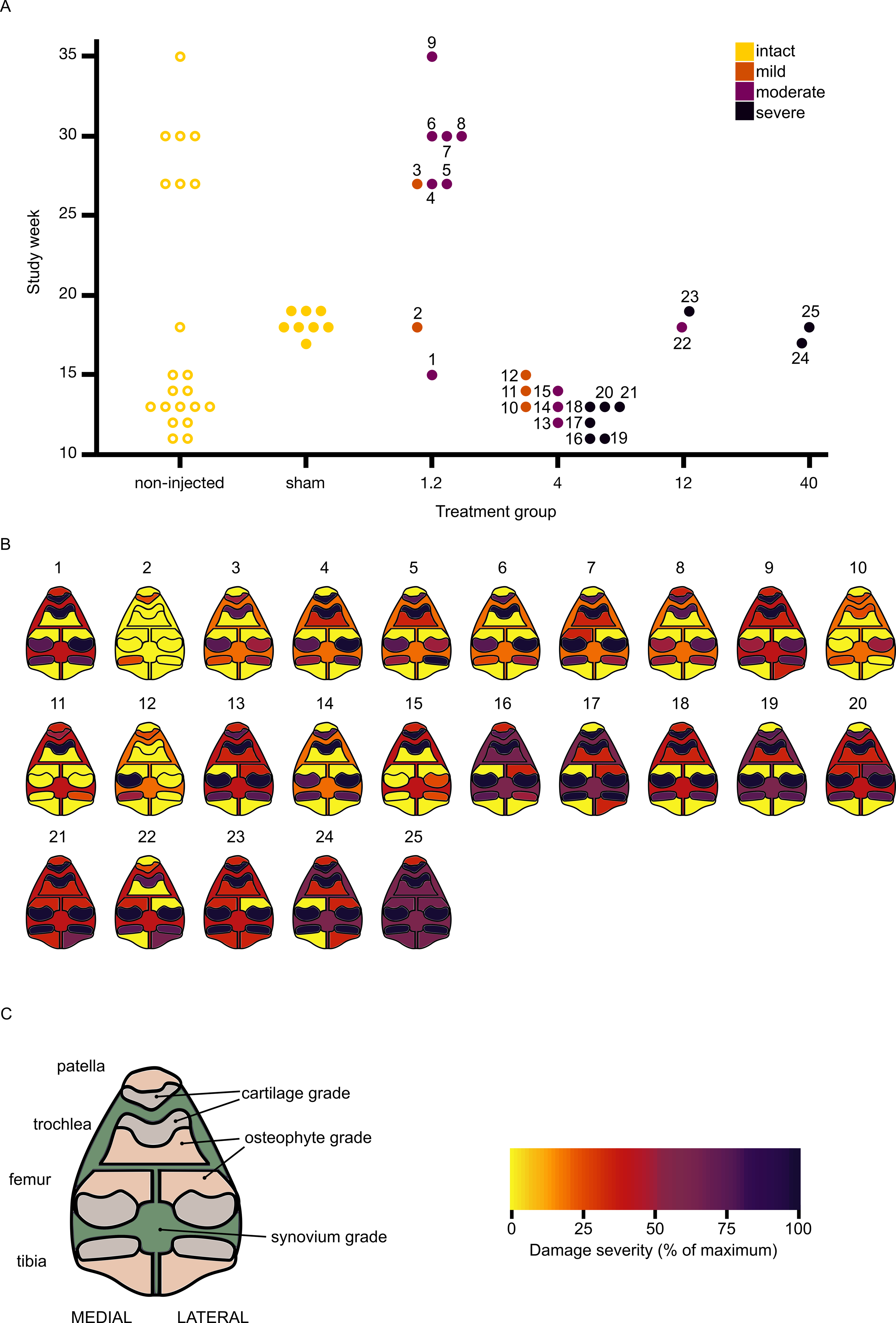

Supplement: S3 Fig — (A) Bilateral hindlimb knees were examined from n = 27 animals and cartilage damage was analyzed according to a global qualitative grading system (intact, mild, moderate, or severe disruption) by a blinded observer. (B) Stylized anatomical models of MIA-injected knees are shown outlining the compartment specific grades for cartilage and osteophytes and a global knee grade for synovial pathology. Each model is numbered to indicate the corresponding data point in A. (C) Legend for the stylized model illustrates the coding of gross data in B. Each point in A represents one knee (n = 54). Injected knees are represented by closed symbols and non-injected knees are represented by open symbols. Sham knees received a 2 ml injection of PBS. The control group consisted of both sham and non-injected knees. (TIF) [file pone.0201673.s003.tif]

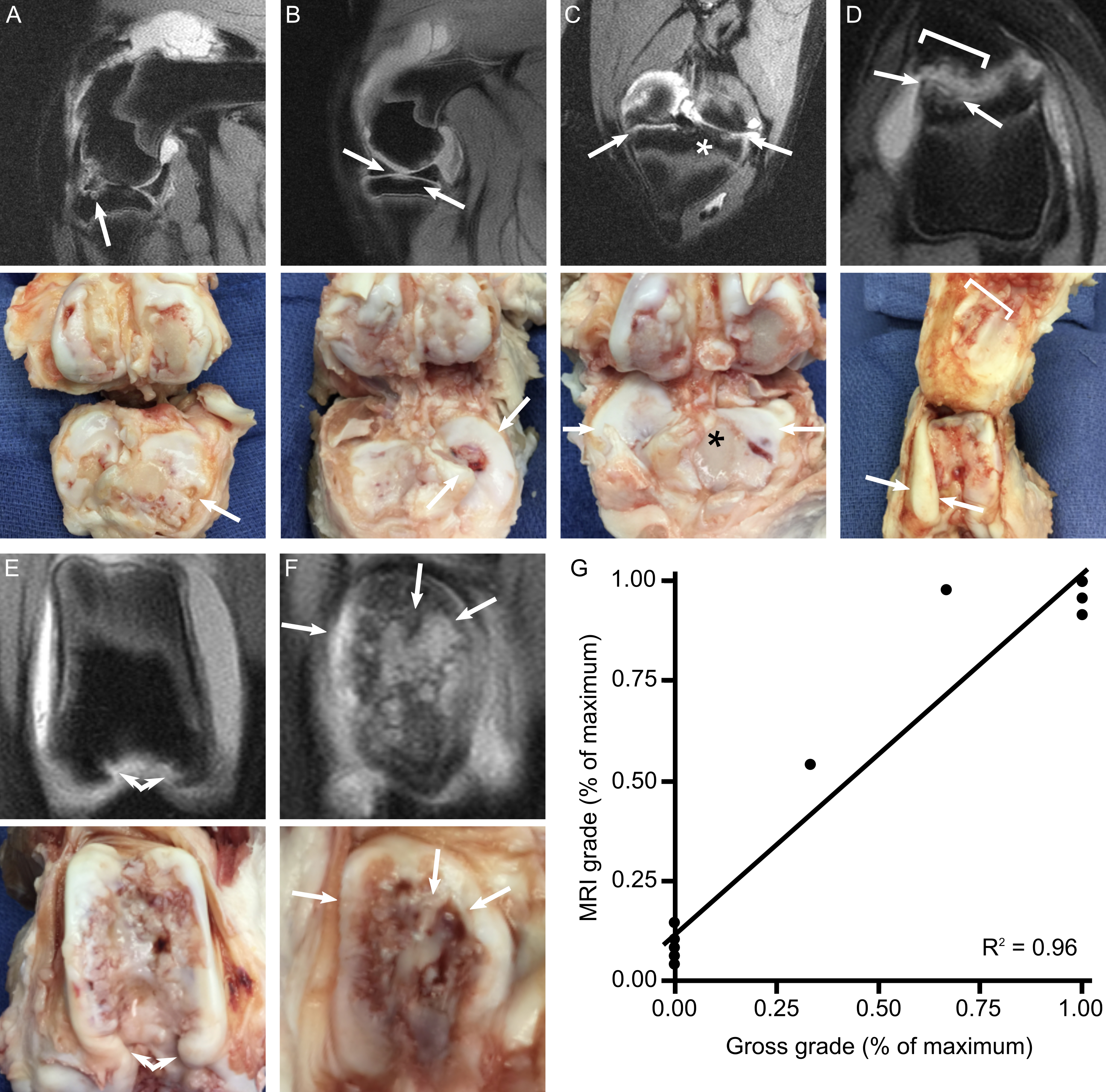

Supplement: S4 Fig — Representative MR images (A–F, top panels) obtained one week prior to gross images (A–F, bottom panels). A total of n = 14 knees were found to have been harvested one week after final MR imaging (bilateral hindlimb knees from 4 mg MIA animals, namely B773, P20, P21, P22, P23, P24, P25). Thinning of articular cartilage overlying the tibial plateau was identified, ranging from partial (arrows, A and B) to complete (asterisk, C). (D) An interlocking patellofemoral erosion revealed a complete grade 4 lesion of patellar cartilage with subchondral erosion (brackets) but not at adjacent trochlea cartilage (arrows, D). (E) Midsagittal invagination of the femoral groove illustrated the effect of friction on thinning damaged cartilage (arrows). (F) MRI revealed isolated fissures and undulating rents in patellar cartilage that were confirmed upon gross inspection (arrows). (G) No significant difference existed between the MRI and gross grades of cartilage damage (P = 0.34). Cartilage grades from all six surfaces of each knee were totaled and divided by the numerical maximum score obtainable (3) to give a normalized grade (percent of maximum). Values were expressed as the mean, ± confidence interval. Statistical significance (P < 0.05) was determined using the paired Student’s t-test (two-tailed) between the two modes of evaluation. (TIF) [file pone.0201673.s004.tif]
