# Supplementary material for: Clinical magnetic resonance-enabled characterization of mono-iodoacetate-induced osteoarthritis in a large animal species
Source: PLoS One. 2018 Aug 3;13(8):e0201673. doi: 10.1371/journal.pone.0201673 (PMC6075758; doi:10.1371/journal.pone.0201673)
Supplement: S1 Table — (DOCX) [file pone.0201673.s005.docx]

**S1 Table. Summary of swine characteristics used in this study.**

| Animal ID | Sex^a^ | Breed | Injection age, weight (mo, kg) | MIA dose (mg) | Injection volume (ml) | Left knee treatment | Right knee treatment | Sacrifice week^b^ |
| --- | --- | --- | --- | --- | --- | --- | --- | --- |
| B544 | female | Yucatan | 9.5, 33.0 | 12.0 | 2.0 | MIA | PBS | 19 |
| B545 | female | Yucatan | 9.5, 32.0 | 0.0 | 2.0 | PBS | PBS | 19 |
| B546 | male | Yucatan | 7.8, 32.0 | 12.0 | 2.0 | PBS | MIA | 18 |
| B548 | female | Yucatan | 9.2, 33.0 | 40.0 | 2.0 | PBS | MIA | 18 |
| B550 | male | Yucatan | 8.7, 30.0 | 40.0 | 2.0 | MIA | PBS | 17 |
| B551 | female | Yucatan | 9.2, 34.0 | 0.0 | 2.0 | PBS | PBS | 18 |
| B771 | male | Yucatan | 8.5, 35.0 | 1.2 | 2.0 | PBS | MIA | 18 |
| B772 | male | Yucatan | 8.5, 39.0 | 4.0 | 2.0 | PBS | MIA | 15 |
| B773 | male | Yucatan | 7.3, 36.0 | 4.0 | 2.0 | MIA | PBS | 13 |
| B774 | female | Yucatan | 7.3, 34.0 | 4.0 | 2.0 | PBS | MIA | 14 |
| B775 | male | Yucatan | 7.6, 36.0 | 1.2 | 2.0 | MIA | PBS | 15 |
| B776 | male | Yucatan | 7.6, 35.0 | 4.0 | 2.0 | PBS | MIA | 14 |
| P20 | female | Yucatan | 6.6, 34.0 | 4.0 | 3.0 | none | MIA | 12 |
| P21 | male | Yucatan | 6.6, 37.0 | 4.0 | 3.0 | MIA | none | 13 |
| P22 | male | Yucatan | 6.6, 31.0 | 4.0 | 3.0 | none | MIA | 12 |
| P23 | female | Yucatan | 6.8, 35.5 | 4.0 | 3.0 | MIA | none | 11 |
| P24 | male | Yucatan | 6.6, 37.5 | 4.0 | 3.0 | none | MIA | 13 |
| P25 | female | Yucatan | 6.5, 35.0 | 4.0 | 3.0 | MIA | none | 11 |
| P26 | male | Yucatan | 6.6, 36.0 | 4.0 | 3.0 | none | MIA | 13 |
| P27 | female | Yucatan | 6.6, 33.5 | 4.0 | 3.0 | MIA | none | 13 |
| P336 | female | Yucatan | 9.8, 37.0 | 1.2 | 3.0 | MIA | none | 27 |
| P338 | female | Yucatan | 9.2, 37.0 | 1.2 | 3.0 | MIA | none | 27 |
| P339 | female | Yucatan | 7.8, 34.0 | 1.2 | 3.0 | none | MIA | 30 |
| P341 | male | Yucatan | 7.8, 34.0 | 1.2 | 3.0 | MIA | none | 35 |
| P342 | male | Yucatan | 7.4, 34.0 | 1.2 | 3.0 | none | MIA | 27 |
| P346 | male | Yucatan | 8.3, 35.0 | 1.2 | 3.0 | MIA | none | 30 |
| P347 | male | Yucatan | 8.6, 35.0 | 1.2 | 3.0 | none | MIA | 30 |

^a^All male animals used in this study were castrated by the supplier.

^b^No animal fell ill during the course of the study and all animals were sacrificed at their respective primary endpoint.
